# Defining blood deserts and access to blood products for 660 million people: a geospatial analysis of eight states in Northern India

**DOI:** 10.1136/bmjgh-2024-015637

**Published:** 2024-10-21

**Authors:** Shreenik Kundu, Alejandro Munoz Valencia, Sargun Kaur Virk, Nikathan Kumar, Anita Gadgil, Joy John Mammen, Nobhojit Roy, Nakul Raykar

**Affiliations:** 1 McGill University Health Centre, Montreal, Quebec, Canada; 2 Program in Global Surgery and Social Change, Harvard Medical School, Boston, Massachusetts, USA; 3 University of Pittsburgh, Pittsburgh, Pennsylvania, USA; 4 Weill Cornell Medical College, New York, New York, USA; 5 Department of Surgery, University of California San Francisco, San Francisco, California, USA; 6 Department of Surgery, WHOCC for Research in Surgical Care Delivery in LMIC, BARC Hospital, Mumbai, Maharashtra, India; 7 Christian Medical College and Hospital Vellore, Vellore, Tamil Nadu, India; 8 University of Global Health Equity, Kigali, Rwanda; 9 Department of Global Public Health, Karolinska Institute, Stockholm, Sweden; 10 Centre for Surgery and Public Health, Brigham and Women's Hospital, Boston, Massachusetts, USA

**Keywords:** Global Health, Blood disorders, Geographic information systems, Surgery

## Abstract

**Introduction:**

Blood transfusion is crucial, but low-income and middle-income countries like India face a severe shortage of banked blood. This study focuses on the Empowered Action Group (EAG) states in India, where healthcare is limited, and health outcomes are poor. Our objective was to assess the blood banking infrastructure and access to blood products in these states.

**Methods:**

We used e-Rakht Khosh, an online platform for blood availability data. We collected data on blood bank locations and stocks from 18 January to 9 February 2022 and used ArcGIS to determine the population residing within 30–60–90 min of a blood bank. Availability ratios were calculated by dividing available blood products by population in these catchment areas. Descriptive analysis characterised availability, and statistical tests evaluated differences across states and over the 4-week period.

**Results:**

806 of 824 blood banks reported data on blood stocks. Our analysis showed that 25.72% of the EAG states’ population live within 30 min of a blood bank, while 61.45% and 92.46% live within 60 and 90 min, respectively.

**Conclusion:**

Blood availability rates were low in the EAG states, with only 0.6 units per 1000 people. Additionally, only 61% of the population had access to blood-equipped facilities within an hour. These rates fell below the standards of the Lancet Commission on Global Surgery (15 units per 1000 population) and the WHO (10 donations per 1000 population). The study highlights the challenges in meeting demand for blood in emergencies due to inadequate blood banking infrastructure.

**WHAT IS ALREADY KNOWN ON THIS TOPIC**

- Blood transfusion is essential for managing various medical conditions, yet there is a significant shortage of banked blood in low-income and middle-income countries like India. India has databases, including the e-RaktKosh platform, which collects nationwide blood transfusion data. Prior to this study, there was limited understanding of how many people in India live with marginal access to blood products.

**WHAT THIS STUDY ADDS**

- This study provides insights into the blood banking infrastructure and blood product availability in India’s Empowered Action Group states using geospatial analysis. It also serves as a proof of principle for employing geospatial analysis to assess blood product availability in other regions or nations with similar challenges. It identifies discrepancies between national blood donation estimates and reported blood product availability, highlighting the need for improved access to blood transfusion services, and addressing blood insufficiency in economically disadvantaged states. The study emphasises the impact of factors like rurality, geographical barriers and limited resources on access to blood and blood products. It reveals both the proximity of populations to blood banks and the inadequacy of accessibility within critical time frames, impacting timely access to emergency transfusion services.

**HOW THIS STUDY MIGHT AFFECT RESEARCH, PRACTICE, OR POLICY**

- Further research should explore the underlying factors contributing to the underestimation of blood collection rates on platforms like e-RaktKosh and develop strategies for accurate reporting. Additionally, more detailed data on blood availability is required to better understand and address disparities in access to different blood products within states and regions.
- The study highlights the urgent need to improve blood transfusion services in economically disadvantaged regions like the EAG states in India. It emphasizes addressing factors such as rurality, geographic barriers, and limited resources that hinder access to blood products. Geospatial analysis proves effective for measuring access by accounting for distance and travel time.
- The study demonstrates the need for data-informed and nationally coordinated blood transfusion services to ensure the availability and safety of blood products. Policy interventions should focus on increasing the overall availability of blood and blood products, aiming for higher donation rates, and reducing wastage.

## Introduction

 Blood transfusion is an essential intervention to manage conditions ranging from trauma to obstetrics to malaria and cancers.^1^ However, banked blood is a scarce resource in low-income and middle-income countries (LMICs). There is a 102 million unit shortage of blood units in LMICs blood banks every year.^2 3^ In India, with a population of more than 1 billion, the estimated need for blood is approximately 26.2 million units of red cells and components, representing about 23% of the worldwide deficit.^3^ In the years 2019–2020, India had a blood donation rate of 29.5 units per 1000 population. This amounted to only 12.5 million units of blood, which is under half of its projected blood need.^4 5^ While it is clear there is a significant unmet need for blood products at a national level, little is known about how many people live with marginal access to any blood products.

The WHO lists blood and blood products as vital medicines and emphasises that well-organised blood transfusion services are required to ensure the availability and safety of blood products.^6^,^8^ On average, India has 2.2 blood banks per 1 000 000 people, but this varies from state to state.^9^ There is significant variation in each blood bank collection rate. Of the 2626 active blood banks country-wide, 25% of them collect almost 66% of the total blood in the country.^10 11^ There is also variation in the location of these blood banks. According to the Indian National Blood Transfusion Council, every district in India needs to have a blood bank; however, approximately 63 of 766 districts do not have one.^12^ Most blood banks are concentrated in urban centres contributing to disparities in blood distribution between urban and rural India.^11^

Blood bank provision is governed by stringent regulations and quality control measures to ensure safety and prevent disease transmission. This includes rigorous testing, proper storage and detailed documentation.^9^ However, these requirements can be challenging for blood banks in low-resource hospitals due to inadequate infrastructure and a shortage of trained staff. The high costs of compliance and logistical difficulties in maintaining supplies can hinder their operations. While regulations are essential for safety, they can restrict the ability of these facilities to provide timely blood products for transfusion. Balancing regulatory standards with the practicalities of rural healthcare is crucial. This may involve contextual guidelines, financial and technical support, and training for local healthcare workers to enhance blood bank management in resource-limited settings.^13^

**Figure 1:**
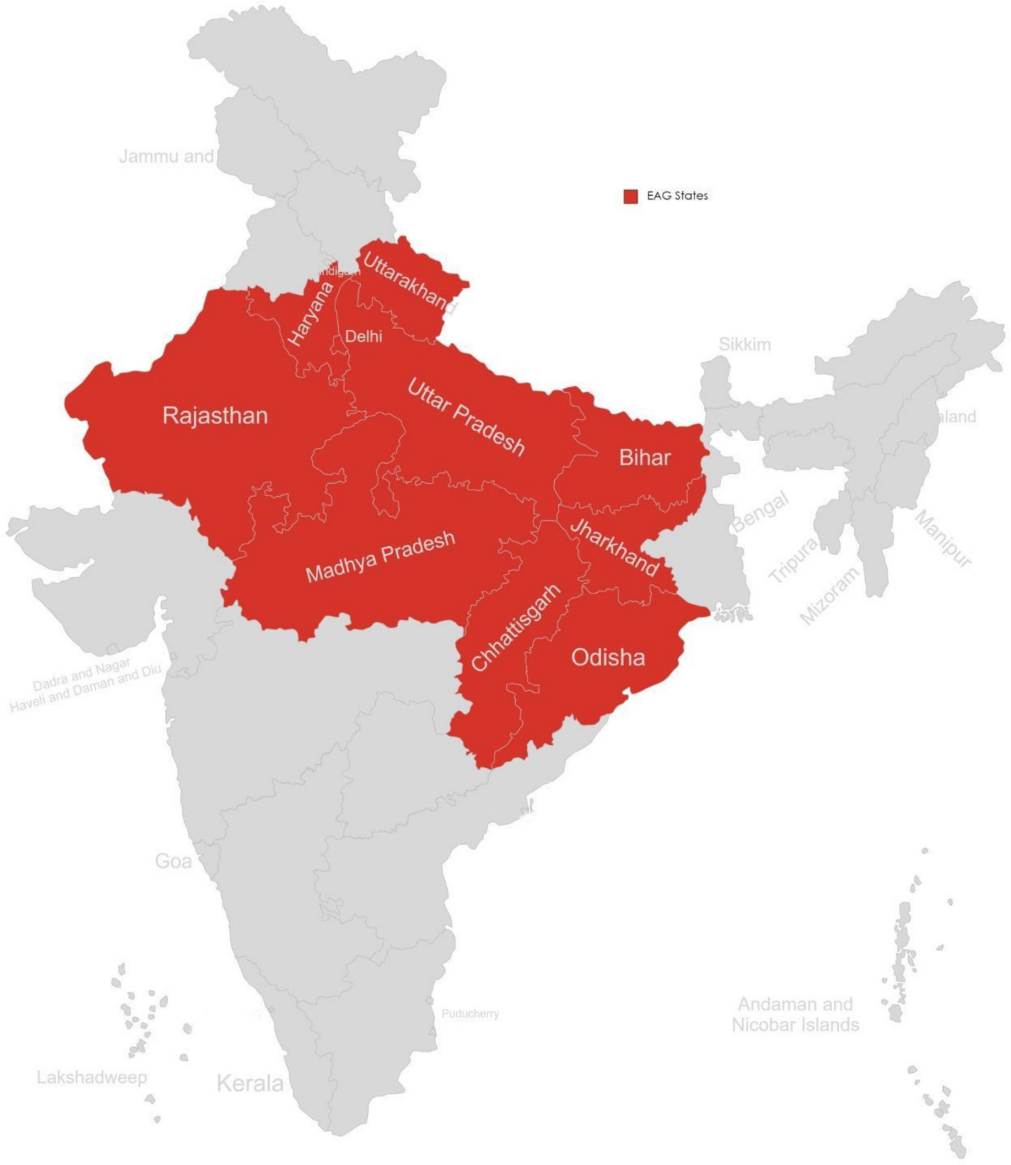
The Empowered Action Group (EAG) states of India.

The Empowered Action Group (EAG) states, as shown in figure 1, are a group of eight Indian states characterised by significant healthcare needs, including higher rates of maternal and infant mortality, widespread poverty, lower literacy rates and underdeveloped infrastructure.^14^ These states occupy approximately 43% of the landmass of India but approximately 46% of India’s total population and 61% of its lowest-income population reside here.^15^ The Ministry of Health and Family Welfare introduced the National Rural Health Mission, which aims to provide adequate healthcare to the population residing in rural areas. The EAG states have been given special attention under this mission to ensure that the areas with the greatest need receive priority.^16^ The EAG initiatives aim to address these disparities through targeted developmental programmes and policies, with a particular emphasis on improving health.^14^ To do this, however, requires a better understanding of the current situation. Therefore, in this study, we aimed to map the blood banking infrastructure and quantify the availability of blood products in the eight EAG states to understand variations in blood availability within this region, as well as define the proportion of the population that lives without any access to blood—that is, the proportion that lives in a ‘blood desert’.

## Methodology

We conducted a multistate, cross-sectional study using data on blood banks in the Indian EAG states including Bihar, Chhattisgarh, Jharkhand, Madhya Pradesh (MP), Orissa, Rajasthan, Uttaranchal and Uttar Pradesh. We employed a modelling approach to assess the availability and accessibility of blood products.

### Databases

We collected blood bank data from the e-RaktKosh data platform and geospatial data from the Environmental Systems Research Institute (ESRI). E-RaktKosh is a web-based data platform maintained by the Ministry of Health and Family Welfare of India, which collects data on the availability (inventory) of blood products across the country.^17^ Established in April 2016, it provides a centralised network of blood banks with publicly available data on blood collection and storage countrywide (n=824). It includes reports from private, governmental, charitable and Red Cross blood banks. We queried the data for the location of all active blood banks and the most recent reports on blood products availability including whole blood, packed red blood cells (PRBCs), fresh frozen plasma and platelet concentrate. We encountered instances where certain blood banks had not updated their records frequently (n=172). For clarity and transparency, we recorded the last updated data available in the database for these blood banks. Moreover, we deleted those (n=18) with no available data during our data collection period. This approach ensured that we used the most recent data available without assuming similar patterns from nearby locations.

We collected data on a weekly basis for 4 consecutive weeks between January and February of 2022. The ESRI India database was used to obtain basÏe-maps and geographic files in accordance with Ministry of Statistics and Programme Implementation data.^18^ And, the population estimates were accessed from the WorldPop Open Population Repository maintained by the WHO—Centre for Humanitarian Data.^19^

### Availability of blood products

We calculated the number of blood product units available per 1000 population living within the 30-min, 60-min and 90-min catchment areas of blood banks. To achieve this, we used the geospatial analysis software ArcGIS Pro to create driving-time areas around each blood bank. These areas were used to determine the geographical areas that likely depend on the nearest blood bank for their blood supply.^20^

The catchment areas for 30, 60 and 90 min of drive time to the nearest blood bank were mapped, assuming that only one blood supplier was available, regardless of the proximity to multiple blood banks, creating non-overlapping catchment areas. We compiled traffic data using historical traffic patterns available in ArcGIS’s geospatial data, which were incorporated into the geospatial analysis to create more accurate drive-time catchment areas. The drive-time analysis accounted for road conditions, rurality and traffic by using historical traffic data. Population data were mapped at a 100-square-metre pixel resolution. We calculated the population per state and catchment area by adding the total number of inhabitants in each of the pixels within the area of analysis. The population estimates used in our analysis aim to calculate the availability of blood for those in need of transfusion. Although the eligibility of blood donors is an important factor for the supply of blood products, our study focuses solely on the availability of blood to the population requiring transfusions. This approach ensures that our analysis addresses the critical issue of access to blood components for all patients who may need them.

Blood product availability at the state level was calculated by aggregating reported availability data from all blood banks located within state boundaries. In view of the prevalence and routine use of whole blood and PRBCs compared with other blood products in this context, these two products were analysed separately. The availability of each blood product per state, as well as comparisons across the eight EAG states for each week and the entire study period, was analysed using Wilcoxon signed-rank and Kruskal-Wallis tests. All statistical analyses were conducted using Stata software, and a significance level of p <0.05 was deemed statistically significant.^21^

### Patient and public involvement statement

This study did not directly involve patients or the public in the design, recruitment or conduct of the study. The research question and outcome measures were informed by the priorities identified through existing data on blood availability and healthcare challenges in the EAG states. Dissemination of results is achieved through publication in open-access journals and presentations at relevant conferences. There were no patient advisers involved in this study.

Since this study is a secondary data analysis, there were no direct participants to receive feedback from.

## Results

In December 2021, e-Rakt Kosh (eRK) reported the existence of 824 blood banks. The distribution of these blood banks was as follows: 98 in Bihar, 84 in Chhattisgarh, 121 in MP, 290 in Uttar Pradesh, 50 in Uttarakhand (UK), 60 in Odisha, 51 in Jharkhand and 70 in Rajasthan. 18 out of the 824 registered blood banks had no available data during the period of our data collection. Data on the availability of reported bloodstock were collected from 806 (97.8%) blood banks, comprising 374 (46.4%) government facilities, 269 (33.4%) private facilities, 16 (0.01%) Red Cross facilities and 145 (18.0%) charitable blood banks, as shown in table 1. Of the 806 banks, 438 (54%) provided daily updates on the number of blood products collected, while 196 (24.3%) provided monthly updates. However, 172 (22%) did not provide regular updates or provided updates at longer than a month’s interval.

**Table 1:** Blood bank characteristics

States	Population	Blood banks	Blood banks per 100 000 population	Govt. blood banks	Private blood banks	Red cross	Charitable blood banks	Govt. blood banks per 100 000	Private blood banks per 100 000	Red cross per 100 000	Charitable blood banks per 100 000
Bihar	128 070 753	96	7.49	37	41	6	12	2.89	3.2	0.47	0.94
Chhattisgarh	30 121 013	83	27.55	26	40	1	16	0.86	1.33	0.003	0.53
Jharkhand	39 862 652	51	12.8	28	18	2	3	0.7	0.45	0.005	0.008
Rajasthan	81 918 609	70	8.55	45	16	0	9	0.55	0.2	0	0.11
Madhya Pradesh	85 837 913	122	14.23	64	41	6	11	0.75	0.48	0.007	0.01
Odisha	47 483 547	58	12.22	52	5	1	0	1.09	0.11	0.002	0
Uttar Pradesh	236 267 158	282	11.94	96	105	0	81	0.41	0.44	0	0.34
Uttarakhand	12 020 584	44	36.62	26	13	0	13	2.16	1.08	0	1.08
Total	661 582 229	806	12.18	374	269	16	145	0.57	0.41	0.002	0.22

Blood banks as a proxy for access to transfusion; population, number of blood banks, blood banks per 100 000 population and blood banks per 100 000 population by type in the 8 target states.

The median number of blood products in stock by state was 2675 (IQR: 1913–5652) PRBCs and 2753 (IQR: 1807–4277) units of whole blood. MP had the highest reported absolute availability with 23 126 PRBC and 22 163 whole blood units, while UK and Odisha had the lowest reported stock availability with 8806 PRBC and 2746 whole blood units and 4063 PRBC and 16 743 whole blood units, respectively.

Week-to-week variations in reported blood stock levels showed significant disparities among different states: for PRBCs, variations ranged from 1.9% in Bihar to 26% in MP, while for whole blood, the variation spanned from 10.9% in Rajasthan to 53% in Bihar.

**Figure 2:**
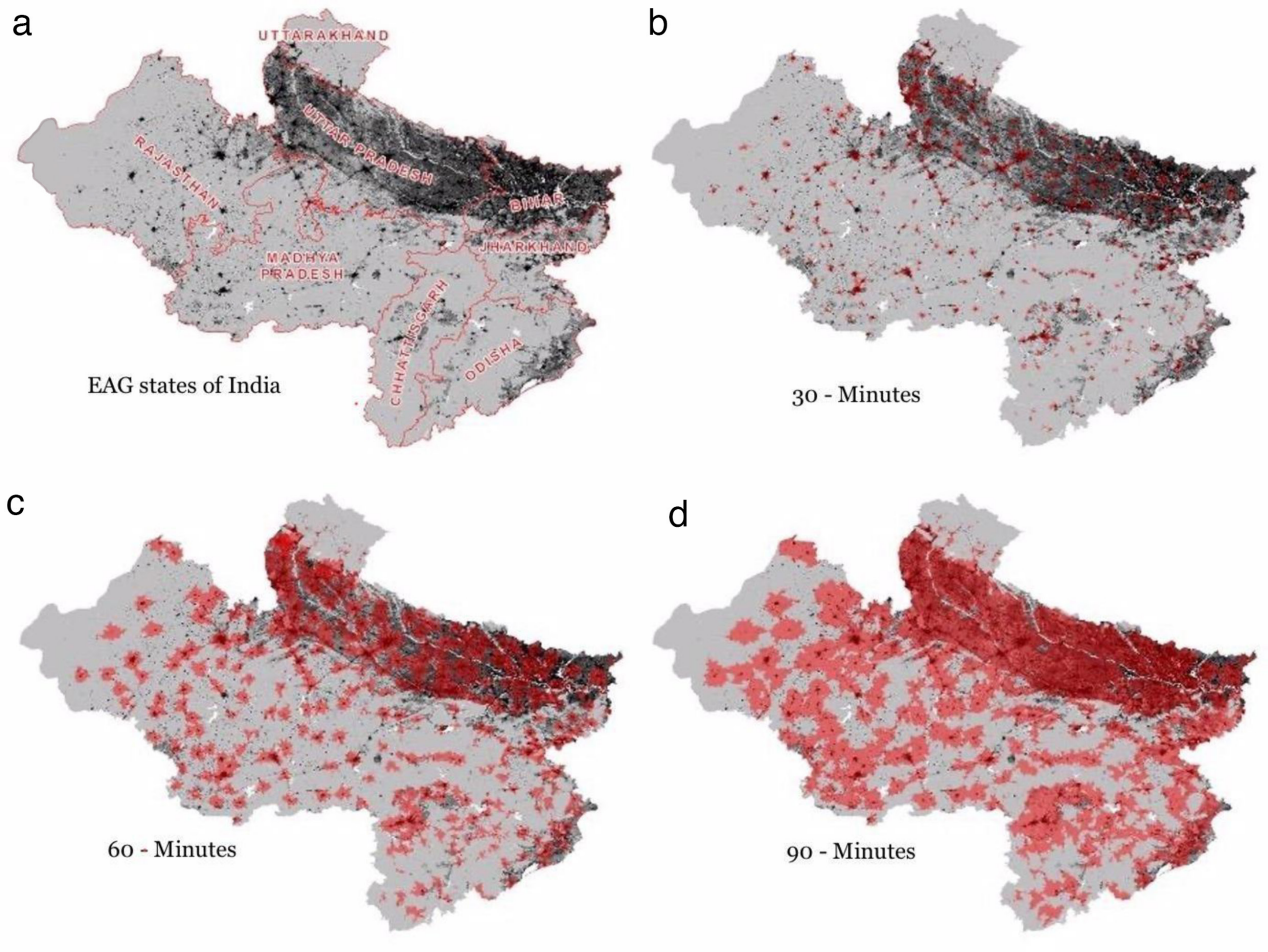
Empowered Action Group (EAG) states population and blood bank coverage areas: (a) geographical boundaries of the eight states, (**b) **30-min drive time areas, (c) 60-min drive time areas and (d) 90-min drive time areas. Dark areas represent regions with higher population densities and red areas represent blood bank coverage within a catchment.

We mapped three distinct catchment areas for each blood bank to depict the geographical areas that could be accessed within a specified time frame. Figure 2 displays the 30-min, 60-min and 90-min drive-time catchment areas to the nearest blood bank. Overlapping populations were only counted once. Within the EAG states, 170 186 835 individuals (25.72%) reside within 30 min of a blood bank, 406 520 732 individuals (61.45%) reside within 60 min, and 611 739 353 individuals (92.46%) reside within 90 min. State-specific details on these catchment areas are provided in table 2. Blood banks in Odisha, Uttar Pradesh and UK have catchment areas that encompass populations from neighbouring states due to the proximity of blood banks to state boundaries. Despite the wide range in coverage at 90 min, with MP at 40% and Jharkhand at 91%, we observed no significant differences in population coverage across states when comparing the 30-min, 60-min and 90-min catchment areas.

**Table 2:** States population and accessibility to blood banks

States	Total population	Population coverage: 30 min (%)	Population coverage: 60 min (%)	Population coverage: 90 min (%)
Bihar	128 070 753	23 283 227 (18.18)	71 750 059 (56.02)	110 051 670 (85.92)
Chhattisgarh	30 121 013	7 179 364 (23.83)	15 936 178 (52.91)	22 867 616 (75.92)
Jharkhand	39 862 652	10 455 992 (26.23)	21 972 935 (55.11)	36 354 526 (91.18)
Rajasthan	81 918 609	18 248 181 (22.27)	37 119 497 (45.31)	62 234 441 (75.96)
Madhya Pradesh	85 837 913	8 515 442 (9.92)	20 875 008 (24.32)	34 724 799 (40.45)
Odisha	47 483 547	16 297 731 (34.32)	37 453 865 (78.87)	65 871 183 (100*)
Uttar Pradesh	236 267 158	80 445 830 (34.05)	187 903 991 (79.53)	257 407 692 (100*)
Uttarakhand	12 020 584	5 761 069 (47.94)	13 509 199 (100*)	22 227 427 (100*)
Total	661 582 229	170 186 835 (25.72)	406 520 732 (61.44)	611 739 353 (92.46)

* Catchment areas include populations from adjacent states.

We calculated the availability of blood products as the number of units per 1000 population for each state, as shown in table 3. Overall, we found that there was a median of 1.42 units per 1000 population (IQR: 1.03–1.85) for individuals living within a 30-min distance from a blood bank, while the availability decreased to 0.63 units (IQR: 0.46–0.78) within 60 min and 0.42 units (IQR: 0.30–0.50) within 90 min, as shown in table 3. It is important to note that only the MP state showed a significant difference in availability rates between different distances to the nearest blood bank. The availability rate ranged from 2.80 per 1000 population for all blood products within 30 min to 0.19 per 1000 population for whole blood and PRBCs within 90 min, with a p value of 0.03.

**Table 3:** Blood product availability by state and catchment area

States	Total blood product Availability for each state	All blood products (30 min)	WB+PRBC (30 min)	All blood products (60 min)	WB+PRBC (60 min)	All blood products (90 min)	WB+PRBC (90 min)	P value
Bihar	0.095	0.53	0.20	0.17	0.06	0.11	0.04	0.92
Chhattisgarh	0.393	1.71	0.50	0.77	0.22	0.53	0.15	0.92
Jharkhand	0.243	0.93	0.46	0.44	0.22	0.26	0.13	0.75
Madhya Pradesh	0.584	2.80	0.65	1.41	0.32	0.84	0.19	0.03*
Odisha	0.343	1.80	0.60	0.77	0.24	0.46	0.14	0.92
Rajasthan	0.274	1.30	0.51	0.59	0.22	0.33	0.12	0.92
Uttar Pradesh	0.369	1.05	0.38	0.45	0.16	0.32	0.12	0.92
Uttarakhand	0.878	1.80	0.50	0.78	0.22	0.47	0.13	0.92
All EAG states	0.332	1.51	0.50	0.68	0.22	0.40	0.13	0.75

All numbers expressed as the number of blood products per 1000 population.

* Statistically significant.

PRBC, packed red blood cells; WB, whole blood.

## Discussion

Timely access to blood and blood products is essential to treat a wide array of human ailments that involve haemorrhage and severe anaemia, ranging from trauma to obstetric emergencies to gastrointestinal bleeding, where patients can suffer from life-threatening haemorrhage within minutes. We found that up to 75% of the population of the EAG states lives effectively without timely access to blood (living beyond 30 min driving distance to a blood bank). When a more lenient measure of timely access to blood is applied (living within 60 min driving distance to a blood bank), almost 40% of the population of the EAG states lives without timely access. However, it is important to note that proximity to a blood bank does not necessarily equate to proximity to a hospital equipped with blood. Our study serves as a proof of principle for employing geospatial analysis to assess blood product availability in other regions or countries with similar challenges.

If a ‘blood desert’ is to be defined as a geographical region without access to blood transfusion, both due to being (1) beyond the bounds of a timely blood transfusion facility and (2) lack of availability of blood at the facilities within reach, *essentially all of the EAG states represent an effective blood desert*. After all, living within an arbitrary, time-bound geographical radius of a blood bank is a rather crude, yet conservative estimate of access to blood availability. Living within these bounds does not confer timely blood access, but living beyond these boundaries almost certainly means a lack of access. Further, the availability of blood for the few patients who live within these 30-min and 60-min boundaries is extremely limited, ranging from 0.68 units/1000 population (within 60 min) to 1.51 units/1000 population (within 30 min), both essentially an order of magnitude below accepted population-based estimates of blood need and well-known thresholds from the WHO and The Lancet Commission on Global Surgery (LCoGS).

There are many reasons why living within a time-bound geographical radius of blood does not automatically confer access to timely blood transfusion. For example, the blood bank facility may not be associated with a hospital or facility that can provide timely transfusion, or presenting to a hospital capable of transfusion with an associated blood bank does not guarantee the presence of equipment, workforce, or systems necessary to diagnose and administer a haemorrhagic condition within an expeditious time frame.^22^

No validated definition exists; however, the Blood Desert Coalition recently discerned blood deserts based on time to a facility.^23^ They defined a ‘blood desert’ as a geographical region with no timely and affordable access to blood components in more than 75% of cases where transfusions are necessary. Similarly, our study’s definition of a blood desert—regions beyond a 30-min drive time to the nearest blood bank—parallels these findings, indicating substantial barriers to timely care. The 30-min threshold is analogous to definitions used in studies on ‘trauma deserts’, which were previously defined as being outside a 5-mile distance to a trauma centre. However, more recent studies suggest that the true ‘desert’ should be based on transport time rather than transport distance.^24^ Factors such as geography, rurality and limited road and transportation infrastructure can significantly impede access to blood facilities. In the world’s poorest communities, including in the EAG states, timely access to a vehicle capable of transport is further limited, and many patients rely on public transportation.

Our findings align with studies conducted in Canada and India, highlighting the critical impact of distance on healthcare outcomes. For instance, a recent study on trauma care in Canada found that travel times greater than 30 min to a trauma centre were associated with a 66% increased risk of death for road traffic accident victims.^25^ Another study in India estimated that the proportion of obstetric emergencies transported by ambulance services was as low as 9.0% in Chhattisgarh, highlighting significant barriers to access in the EAG states.^26^ This demonstrates the importance of timely access to care, which is similarly critical for patients needing blood transfusions. The parallels between trauma care and blood transfusion services emphasise the need for improved accessibility to healthcare facilities in rural and underserved regions.

We observed significant regional variations in blood product inventory. For instance, MP had the highest stock of 23 126 PRBCs and 22 163 whole blood units, in contrast to UK and Odisha with much lower stocks. This disparity in stock availability demonstrates a significant variation in the use of and demand for different blood products across regions, reflecting differing medical practices and needs. Since 2010, the WHO has advocated for a minimum donation rate of 10 donations per 1000 population.^27^ Similarly, in 2015, the LCoGS proposed a target of 15 units per 1000 population.^28^ Recently, Robert *et al* conducted a study that aimed to estimate the global supply of blood products in various countries.^3^ In lieu of the current practice, the authors advocated for a target range of 30–40 donations per 1000 population.^3 29^ Mammen *et al* reported that the average yearly clinical requirement for blood per hospital bed in India was 9.2 units per 1000 population. Our results demonstrate that overall availability in the EAG states is incredibly poor, at 0.68 units/1000 population at a 60-min radius. Unfortunately, this paltry figure is not the lower bound, as the availability rate of blood products ranged from 1.4 units per 1000 population in the state of MP to 0.17 units per 1000 population in the state of Bihar.^30^

In the context of blood availability, it is important to distinguish between blood donation rates and facility-reported blood inventory. The WHO’s recommended 10 donations per 1000 population represents the number of donations made, not the number of usable blood units available in inventory. The actual blood inventory is subject to various factors, including the characteristics of donors (age, health status, epidemiology), and wastage from logistical challenges, product expiry and adherence to quality standards. Therefore, while blood donation is a critical component of blood supply, our study focuses on facility-reported blood inventory, which indicates the number of blood units available for transfusion at any given time.

The insufficiency of the blood supply in the EAG states highlights the need to understand the underlying indications for blood transfusion, which vary by locale and facility. Common indications include polytrauma, obstetric emergencies, severe anaemia and other surgical procedures.^30^ According to recent studies in India and Tanzania, the demand for blood transfusions is highest in medical specialties, followed by surgery, obstetrics and gynaecology, and paediatrics.^31^ These variations reflect the similar medical needs and practices across low-resource regions, influencing blood product demand and availability.

Commonly reported measures of blood availability are inadequate in assessing availability and sufficiency of blood for a given population. Governments and reporting agencies should, instead, incorporate some aspect of geospatial access into assessments and, at a minimum, report time-bound geography-based population access to blood transfusion-capable facilities, as well as blood product availability to these specific populations. For example, the WHO defines the whole blood donation rate as a proxy measure of a country’s overall blood supply, which can be enhanced by encouraging voluntary non-remunerated blood donations throughout the year.^29^ It is important to note that various factors such as infections, resource limitations and inadequate component preparations can delay the process of blood becoming available for transfusion, altering the life cycle of blood from donation to availability. Moreover, blood products require special processing and storage conditions with specific shelf-lives based on components as well as handling conditions. Expiration and wastage of blood are not insignificant within most blood systems, with rates ranging from 5% to 15%.^32^ Therefore, national blood donation rates may overestimate the actual number of blood products available in a country and fail to acknowledge the tremendous variation between regions or the depth of scarcity in the most rural, remote or socioeconomically disadvantaged areas.

As such, blood availability based on geospatial access to blood banks or transfusion-capable facilities can provide much more nuanced insights that can drive further action. For example, while the overall population that lives within 60 min of a blood bank for the EAG states is 61%, it ranges to as low as 24% in MP, which has a 1.41 blood product per 1000 population availability for this population, to 100% in UK, which has a 0.78 blood product per 1000 population availability for this population. As such, MP would benefit from measures to improve geographical access, such as increasing distribution of blood centres and transfusion-capable facilities in addition to measures to increase overall blood availability. UK requires a different approach, emphasising the improvement of sufficiency to meet its specific blood supply needs. To enhance the effectiveness and reliability of the blood bank system, it is recommended that all blood banks, particularly the 172 (22%) that currently do not provide regular updates or only update at intervals longer than a month, should aim to provide at least monthly, if not daily, updates on the number of blood products collected. This practice has already been adopted by 54% of the banks providing daily updates and 24.3% providing monthly updates. This ensures better transparency, resource management and responsiveness to demand. Governments and national blood transfusion authorities should track and report regional variation in blood availability with a specific focus to identify areas of blood unavailability, or blood deserts.

### Limitations

A notable limitation in our study is the varied reporting frequencies among blood banks. Rates of blood collection reported on the eRK platform are observed to be generally lower than those reported by other blood banks. Our study relies on self-reported data from the e-RaktKosh platform, which may be subject to desirability bias and under-reporting. This limitation affects the accuracy of the reported blood inventory and availability. Additionally, through the experience of one author on this manuscript (JM) with extensive blood banking experience in India, blood banks frequently under-report blood stocks on the eRK platform for a variety of reasons, including technical difficulties with the eRK system, inadequate training and awareness among blood bank personnel, and limited accountability for precise reporting.^33^ Conversely, eRK figures likely do not represent stores that are unavailable based on expiration or wastage and may be over-representations of availability. Nonetheless, the eRK platform is the official public reporting mechanism established by the national blood banking authorities and represents the government’s official estimates of blood stores and our analysis serves as a baseline measure of blood availability based on these estimates. Future studies should aim to validate self-reported data with independent audits to improve reliability.

We recognise that time to transfusion would be a better metric; however, in the absence of these data, we chose distance to blood banks as a conservative proxy for time to transfusion, which represents the best-case scenario in these settings. Using this prevents our model from overstating our results. The reality of the situation is that blood availability in this region is likely much worse than what we have found in this study. Additionally, while the proximity to blood banks is crucial, the ability to donate blood also significantly impacts the overall blood supply and was not analysed in this study. This would provide valuable insights into the sufficiency of hospitals within the region. We also acknowledge that our study does not include data on whether blood banks have additional infrastructure needed to care for patients. Future research should consider the operational capabilities of blood banks and the logistics of blood donation to provide a more comprehensive assessment of access to transfusion services.

Our estimates of driving distance are based on average driving times using ArcGIS geospatial analysis software and may not reflect seasonal variations in road conditions or traffic patterns. We highlighted the varying availability of blood products across the EAG states in India, though do not account for the rural–urban distribution of each state, also an important factor influencing access to healthcare services, including blood transfusions. States such as Uttar Pradesh, Chhattisgarh, Jharkhand and Odisha have significant rural populations which may face more challenges in accessing limited blood products than states with relatively higher urban populations. Further studies should benefit from taking these limitations into account, though we believe that the first step in improving access to blood availability is national-level reporting of *blood unavailability*, which begins with the relatively simple geospatial analysis described in this manuscript.

## Conclusion

A healthcare system without timely access to blood and blood products is incomplete. This study assessed the blood banking infrastructure and access to blood products for transfusion in the underserved EAG states of India. Most estimated blood availability rates were low, 0.6 units per every 1000 people in the heartland of India and only 61% of the population can access a blood-equipped facility within an hour. Blood product availability rates were significantly lower than the LCoGS and WHO recommendations. Without sufficient blood, existing blood banking infrastructure will not function at its optimal capacity. Addressing this gap will require a multisectoral approach that may include facilitating the transfer of blood products between facilities, increasing blood donations and exploring alternative transfusion practices. Timely access to blood should be a key priority for the Indian EAG states and other countries to ensure complete and effective healthcare delivery.

## Data Availability

Data are available in a public, open access repository. Data are available upon reasonable request.
